# Pain management of nalbuphine and sufentanil in patients admitted intensive care unit of different ages

**DOI:** 10.1186/s12873-022-00592-x

**Published:** 2022-03-26

**Authors:** Kaiqiang Ji, Xiaoying Gong, Ting Luan, Xiaopeng Gao, Bin Zang

**Affiliations:** Department of Critical Care Medicine, Shengjing Hospital of China Medical University, No.36 Sanhao Street, Shenyang, China

**Keywords:** Nalbuphine, Sufentanil, Analgesia, Sedation, Intensive care unit

## Abstract

**Background:**

Pain relief for patients in the intensive care unit (ICU) can improve treatment outcomes and reduce the burden on doctors and nurses. This study aims to report the clinical analgesic and sedative effects of nalbuphine and sufentanil on ICU patients.

**Methods:**

This study retrospectively analyzed the medical records of 87 critically ill patients who received nalbuphine or sufentanil infusion in the ICU, including demographic data, diagnosis, Acute Physiology and Chronic Health Evaluation (APACHE) II, Critical Care Pain Observation Tool (CPOT), Richmond Agitation-Sedation Scale (RASS), systolic and diastolic blood pressure, heart rate and blood oxygen saturation (SpO_2_). The primary outcomes of this study were CPOT and RASS scores. The secondary outcomes were hemodynamic changes, including systolic blood pressure, diastolic blood pressure, heart rate, and SpO2. The adverse events recorded during pain management, such as hypoxemia, respiration depression and bradycardia, were also collected and analyzed.

**Results:**

None of the patients in both groups experienced episode of hypoxemia, respiration depression and bradycardia. However, age-stratified analyses showed that nalbuphine has a better analgesic effect than sufentanil for patients aged ≤ 60 (*P* < 0.05). In contrast, sufentanil showed a better analgesic effect than nalbuphine for patients aged > 60 ( *P* < 0.05). Furthermore, nalbuphine has a significantly better sedative effect than sufentanil for patients aged ≤ 60 (*P* < 0.05).

**Conclusion:**

ICU patients of different age groups may be suitable for different analgesics. For patients under the age of 60, nalbuphine has better analgesia and sedation than sufentanil, and does not cause respiratory depression and drastic hemodynamic changes.

## Background

Pain management is a major public health problem in social, economic and clinical fields, especially for patients in the intensive care unit (ICU) [1]. Most patients in the ICU experience moderate to severe pain intensity, and pain experience and treatment-related anxiety increase physical and emotional distress, which may interfere with wound healing, recovery and even increase mortality [2–4]. The appearance of pain is not only related to the patient’s condition, but also caused by the nursing care, such as deep breathing, coughing exercises, turning, drain removal, and endotracheal suctioning [5, 6]. Hence, alleviating pain is an essential clinical practice in ICU when caring for critically ill patients. Analgesics and sedatives can be effectively used by assessing the pain intensity of critically ill patients, which will have a positive impact on ICU clinical practice [7]. Clinical practice guidelines recommend the use of intravenous opioids as the first-line drug class for perioperative pain management and pain relief in ICU patients [8]. However, the top up demand use of opioids is not always used, mainly due to concern of side effects such as respiratory depression.

Despite the advance in health care, sedation and analgesia are still important aspects of patient care on the intensive care unit. Intravenous opioids are most commonly used to manage the distress caused by pain in ICU patients. Although there are international differences in the prescription of sedative and analgesic drugs, the opioids most commonly used for analgesia are morphine, fentanyl, sufentanil, and nalbuphine [9, 10]. Below table shows the commonly used opioid analgesics in ICU patients (Table 1). Although morphine is the most common opioid, morphine can induce a variety of adverse events, such as vomiting, itching, nausea, drowsiness, urinary retention, constipation and respiratory depression. In contrast, sufentanil is another μ-opioid agonist, which has been commonly used in pediatric and adult patients as an auxiliary drug in anesthesia for decades to treat moderate to severe pain [11]. Due to the high lipophilic properties, sufentanil has a rapid onset and offset time after intravenous injection, with a half-life of about 15 min [12]. Several clinical trials have showed that sufentanil has excellent pain control in patient-controlled management of acute postoperative pain, and the most typical adverse events of sufentanil are nausea, vomiting, dizziness, and respiration depression [13, 14].

**Table 1:** Common opioid analgesics for ICU patients

Drug	Mechanism of action	Equivalent dose	Onset	Duration of analgesia	Common adverse reactions
Morphine	μ-opioid agonist (also with κ-opioid and δ-opioid agonist effects)	10 mg	15–60 min	4–5 h	Respiration depression, Nausea, Vomiting, Drowsiness, Constipation, Hypotension, Urinary retention, Itching
Fentanyl	μ-opioid agonist (also with κ-opioid agonist effects)	0.1 mg	< 1–2 min	1–1.5 h	Respiration depression, Nausea, Constipation, Skeletal-muscle rigidity
Sufentanil	μ-opioid agonist (also with κ-opioid agonist effects)	0.02 mg	within a few minutes	1–1.5 h	Respiratory depression, Nausea, Dizziness, Vomiting, Itching, Skeletal-muscle rigidity
Nalbuphine	μ-opioid antagonist (also with κ-opioid agonist effect)	10 mg	2–3 min	3–6 h	Nausea, Drowsiness, Sweating, Dry mouth, Dizziness. (nearly no psychotomimetic side effects)

Nalbuphine is a powerful synthetic opioid agonist–antagonist analgesic. Studies have shown that nalbuphine can bind to the μ-opioid and κ-opioid receptors in the medulla and cerebral cortex, thereby providing effective analgesic [15–18]. The analgesic effect of nalbuphine is equivalent to that of morphine, and its onset time is similar to that of fentanyl [19, 20]. The short onset time means that continuous infusion of nalbuphine can sustain the desired analgesic effect. Although nalbuphine shows the same degree of respiratory depression as the dose of morphine, nalbuphine has a ceiling effect on respiratory depression, that is, when the dose of nalbuphine is greater than 30 mg/70 kg, the respiration depression effect will no longer increase with the increase of the dose [21]. These properties make nalbuphine considered to be a more ideal and safer analgesic, and it is widely used in pediatric and gynecological surgery [22, 23].

It is well known that pain management in the ICU has a great impact on short- and long-term outcomes. Although nalbuphine and sufentanil have been used for analgesia in many operations, the difference between sedation and analgesia for ICU patients is still unclear. Both under-sedation and over-sedation may put critically ill patients at high risk of prolonged mechanical ventilation, longer ICU and hospital stay, organ system failure, and prolonged reintubation rates. Thus, the aim of this study was to compare the analgesic and sedative effectiveness and the impact on analgesia/sedation-related adverse events between nalbuphine and sufentanil in patients admitted to ICU.

## Methods

### Study population

From 2018 to 2019, critically ill patients who received nalbuphine or sufentanil in the ICU were included. All patients were intubated in the ICU. Patients who allergic to nalbuphine and sufentanil, pregnant women and lactating women were excluded. In addition, patients that were readmitted to the ICU were excluded, that is, only the first admission to the ICU was considered for this study. A total of 78 patients were included in this study. A power analysis was also conducted to estimate the included sample size. The estimated sample size was based on a power analysis for 5 repeated measures of variance using an estimated medium effect size (*f* = 0.25), an alpha level of 0.05, and a power of 0.8. After analysis, there should be at least 22 people in each group. Therefore, the patients included in this study are sufficient to achieve statistical significance. The clinical data of each enrolled patient were obtained from medical record review of the ICU audit database, including demographic data, reasons for admission to the ICU, type of anesthesia, Acute Physiology and Chronic Health Evaluation (APACHE) II score, Critical Care Pain Observation Tool (CPOT) and Richmond Agitation-Sedation Scale (RASS). In addition, hemodynamic parameters during anesthesia were collected and analyzed, including systolic and diastolic blood pressure, heart rate and blood oxygen saturation (SpO_2_).

The primary outcomes were the analgesic and sedative effects of sufentanil and nalbuphine, which were evaluated by CPOT and RASS, respectively. The four items of CPOT include facial expression, body movements, limb muscle tension, and compliance with ventilator (intubated patients) or vocalization (non-intubated patients). The SPOT score of each item is 0–2 [24]. The higher the score, the higher the level of pain. A SPOT score > 2 is usually considered the presence of pain. The sedation effect of the RASS score, ranging from -5 to 4 points, reflects the change in the patient’s sedation level from deep sedation to high restlessness [25]. A RASS score of -1 to 2 is considered the proper level of sedation. The secondary outcomes were hemodynamic changes (systolic blood pressure, diastolic blood pressure, heart rate, and SpO2) and adverse events in ICU (hypoxemia, respiration depression and bradycardia). Hypoxemia is considered significant when the patient’s SpO_2_ < 90% lasted for ≥ 5 s. It was considered to have respiratory depression when the patient experiences end-tidal CO_2_ > 50 mmHg, respiratory rate < 6 breaths/minute, or airway obstruction with cessation of gas exchange at any time. Bradycardia is defined as the patient had a reduction in heart rate to 60 beats/min during infusion with sufentanil and nalbuphine, while arterial is defined as a decrease in systolic blood pressure < 90 mmHg.

### Patient and public involvement

Patients or the public were not involved in the study design, collection, analysis, and interpretation of data or in the writing of this manuscript.

### Statistical analysis

Statistical analyses were performed using SPSS software version 22.0 (IBM, Armonk, NY, USA). Frequency and percentage were summarized for categorical variables. Continuous variables were presented as the mean ± standard deviation (SD) or median with inter-quartile range (IQR). Pearson's chi-squared test was used to analyze categorical variables. The generalized estimating equation (GEE) was used to compare the reduction in pain at different time points between the sufentanil group and the nalbuphine group. In addition, the Student t test was used to analyze the differences between groups (age, weight, systolic blood pressure, diastolic blood pressure, heart rate and SpO_2_). The Mann–Whitney U test was performed to compare the two groups in the hemodynamic parameters, APACHE II, CPOT, and RASS.

## Results

### Demographic and clinical characteristics

The demographic characteristics of the 78 ICU are presented in Table 2. The mean age was 53.36 ± 2.15 years, and 60.3% of the patients were female (39.7% were male). The average weight of the patients was 64.63 ± 1.06 kg. The mean baseline scores of CPOT and RASS were 3.42 ± 0.15 and 1.54 ± 0.09, respectively. The median baseline APACHE II score was 10 (IRQ, 6–17). The mean systolic blood pressure and diastolic blood pressure of the patients were 126.08 ± 2.31 mmHg and 73.28 ± 1.40 mmHg, respectively. The mean heart rate and SpO_2_ were 96.15 ± 2.53 bpm and 96.92 ± 0.62%, respectively. Among the 78 patients, 38 patients received sufentanil and 40 patients received nalbuphine. There was no significant difference in pain intensity assessed by CPOT between the two groups (3.42 ± 0.02 vs. 3.43 ± 0.21, *P* = 0.990). In addition, the sedation/restlessness assessed by RASS was not significant different between groups (1.61 ± 0.13 vs. 14.8 ± 0.14, *P* = 0.493). There were no significant differences between the two groups with respect to systolic blood pressure (128.21 ± 3.43 mmHg vs. 124.05 ± 3.12 mmHg, *P* = 0.371), diastolic blood pressure (71.26 ± 2.07 mmHg vs. 75.20 ± 1.88 mmHg, *P* = 0.162), heart rate (99.71 ± 3.85 bpm vs. 92.78 ± 3.27 bpm, *P* = 0.172) and SpO_2_ (97.34 ± 0.62% vs. 96.52 ± 1.05%, *P* = 0.510).

**Table 2:** Baseline demographics of patients admitted to intensive care units

	**Total *N* = 78**	**Sufentanil *N* = 38**	**Nalbuphine *N* = 40**	***p*-value**^a^
Gender				0.962
Male	31 (39.7%)	15	16	
Female	47 (60.3%)	23	24	
Age (year)	53.36 ± 2.15	55.05 ± 3.32	51.75 ± 2.77	0.446
Weight (kg)	64.63 ± 1.06	63.79 ± 1.64	65.43 ± 1.37	0.444
APACHE II	10 (6, 17)	13 (8, 18)	8.5 (5, 14)	0.072
Disease entity				0.214
Infectious disease	16 (20.5%)	9 (23.7%)	7 (17.5%)	
Trauma and accidental injury	26 (33.3%)	13 (34.2%)	13 (32.5%)	
Neoplasms	10 (12.8%)	3 (7.9%)	7 (17.5%)	
Inflammatory disease	5 (6.4%)	3 (7.9%)	2 (5.0%)	
Digestive systems	7 (9.0%)	6 (15.8%)	1 (2.5%)	
Cardiovascular system	3 (3.8%)	0 (0.0%)	3 (7.5%)	
Respiratory disease	2 (2.6%)	1 (2.6%)	1 (2.5%)	
Others	9 (11.5%)	3 (7.9%)	6 (15.0%)	
CPOT	3.42 ± 0.15	3.42 ± 0.22	3.43 ± 0.21	0.990
RASS	1.54 ± 0.09	1.61 ± 0.13	1.48 ± 0.14	0.493
SBP (mmHg)	126.08 ± 2.31	128.21 ± 3.43	124.05 ± 3.12	0.371
DBP (mmHg)	73.28 ± 1.40	71.26 ± 2.07	75.20 ± 1.88	0.162
HR (bpm/min)	96.15 ± 2.53	99.71 ± 3.85	92.78 ± 3.27	0.172
SpO_2_ (%)	96.92 ± 0.62	97.34 ± 0.64	96.52 ± 1.05	0.510

*Abbreviation: APACHE II* Acute Physiology and Chronic Health Evaluation, *CPOT* Critical Care Pain Observation Tool*, RASS* Richmond Agitation-Sedation Scale*, HR* heart rate, *SpO*_*2*_ oxygen saturation, *SBP* systolic blood pressure, *DBP* diastolic blood pressure

^a^Mann-Whitney test was used for continuous variables, and Fisher’s exact test was used for categorical variables. Data were presented as mean ± standard error (SE) or median with inter-quartile range (IQR)

### Analgesic and sedative effects of sufentanil and nalbuphine on ICU patients

**Fig. 1:**
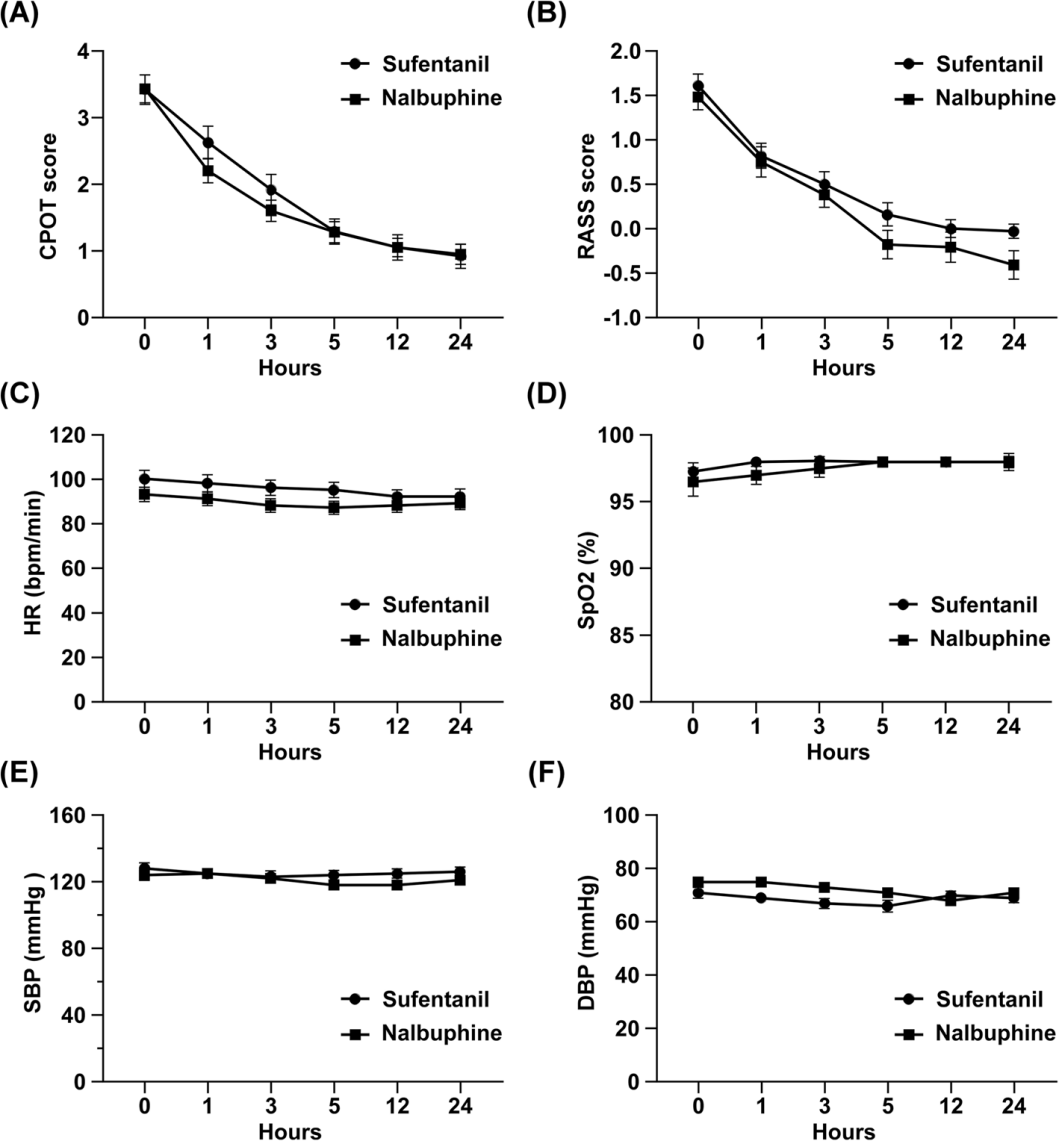
Patients features in the ICU during nalbuphine or sufentanil infusion. (**A**) Pain intensity in ICU patients receiving nalbuphine or sufentanil at different time points (mean ± SD). Pain intensity was evaluated by CPOT. There was no significant difference between groups (GEE, *P* > 0.05). (**B**) Sedation/restlessness intensity in ICU patients receiving nalbuphine or sufentanil at different time points. Sedation/restlessness intensity was evaluated by RASS. Nalbuphine showed a better sedative effect than that of sufentanil (GEE, *P* = 0.037) (**C**) Heart rate of ICU patients at different time points during nalbuphine or sufentanil infusion. There was no significant difference between groups (GEE, *P* > 0.05). (**D**) SpO2 of ICU patients at different time points during nalbuphine or sufentanil infusion. No significant difference was observed between groups (GEE, *P* > 0.05). (**E**) SBP and (**F**) DBP of ICU patients receiving nalbuphine or sufentanil at different time points. Data were expressed as mean ± SD. The *P*-value was calculated using the generalized estimating equation method. *Abbreviation*: *CPOT* Critical Care Pain Observation Tool, *RASS* Richmond Agitation-Sedation Scale, *SpO*_*2*_ oxygen saturation, *SBP* systolic blood pressure, *DBP* diastolic blood pressure

Complete sets of CPOT and RASS scores during anesthesia were collected and analyzed. Table 3 shows the analgesic and hemodynamic parameters of patients receiving sufentanil or nalbuphine at different time points. After anesthesia, the CPOT (Fig. 1A) and RASS (Fig. 1B) scores in both sufentanil and nalbuphine groups gradually decreased (GEE, *P* < 0.001). After 3 h, nalbuphine can effectively reduce the pain intensity (CPOT < 2), and sufentanil also reduced pain intensity after 5 h of infusion. There was no significant difference in analgesic effects between the nalbuphine and the sufentanil groups (GEE, *P* > 0.05). On the other hand, the sedative effect of nalbuphine was significantly better than that of sufentanil (GEE, *P* = 0.037). This result shows that both sufentanil and nalbuphine are effective for analgesia and sedation in ICU patients.

**Table 3:** Patients admitted to intensive care units receiving sufentanil or nalbuphine at each time point

	**0 h**	**1 h**		**3 h**	**5 h**	**12 h**	**24 h**	***p*-value**^a^
CPOT								
Sufentanil	3.42 ± 1.37	2.63 ± 1.50		1.92 ± 1.44	1.29 ± 1.16	1.05 ± 1.13	0.92 ± 1.09	< 0.001
Nalbuphine	3.43 ± 1.36	2.20 ± 1.16		1.60 ± 1.03	1.28 ± 1.04	1.05 ± 0.89	0.95 ± 0.92	< 0.001
*p*-value^b^	0.990	0.157		0.260	0.954	0.991	0.897	
RASS								
Sufentanil	1.61 ± 0.79	0.82 ± 0.87		0.50 ± 0.86	0.16 ± 0.79	0.00 ± 0.62	-0.03 ± 0.50	< 0.001
Nalbuphine	1.48 ± 0.88	0.75 ± 1.10		0.38 ± 0.90	-0.18 ± 1.01	-0.21 ± 1.06	-0.41 ± 1.02	< 0.001
*p*-value^b^	0.493	0.771		0.533	0.110	0.304	0.040	
SBP (mm/Hg)								
Sufentanil	128.21 ± 3.43	124.97 ± 3.04		123.45 ± 3.61	123.84 ± 2.91	124.51 ± 2.76	126.42 ± 2.90	0.912
Nalbuphine	124.05 ± 3.12	125.08 ± 2.70		121.95 ± 2.47	118.25 ± 2.37	118.13 ± 2.48	121.18 ± 2.66	0.286
*p*-value^b^	0.371	0.980		0.731	0.139	0.089	0.186	
DBP (mm/Hg)								
Sufentanil	71.26 ± 2.07	69.34 ± 1.53		66.87 ± 1.85	65.92 ± 2.23	69.89 ± 1.63	69.44 ± 1.73	0.418
Nalbuphine	75.20 ± 1.88	75.45 ± 1.68		73.05 ± 1.45	71.13 ± 1.54	68.46 ± 1.44	70.67 ± 1.55	0.010
*p*-value^b^	0.162	0.009		0.010	0.056	0.511	0.599	
HR (bpm/min)								
Sufentanil	99.71 ± 3.85	98.26 ± 3.90		95.89 ± 3.49	94.58 ± 3.41	92.27 ± 3.08	92.25 ± 3.47	0.580
Nalbuphine	92.78 ± 3.27	91.13 ± 3.05		88.28 ± 3.09	86.70 ± 2.91	88.31 ± 3.03	89.28 ± 2.82	0.766
*p*-value^b^	0.172	0.151		0.105	0.082	0.362	0.507	
SpO_2_ (%)								
Sufentanil	97.34 ± 0.64	97.87 ± 0.34		98.08 ± 0.31	97.79 ± 0.37	98.16 ± 0.32	98.00 ± 0.64	0.872
Nalbuphine	96.52 ± 1.05	97.43 ± 0.67		97.51 ± 0.64	98.10 ± 0.32	98.41 ± 0.28	97.72 ± 0.42	0.296
*p*-value^b^	0.510	0.561		0.430	0.525	0.562	0.709	

*Abbreviation: CPOT* Critical Care Pain Observation Tool*, RASS* Richmond Agitation-Sedation Scale*, SBP* systolic blood pressure*, DBP* diastolic blood pressure*, HR*, heart rate*, SpO*_*2*_*,* oxygen saturation

Data were presented as mean ± standard error (SE) or median with inter-quartile range (IQR). Bold indicates a statistically significant difference with a p-value less than 0.05

^a^The *P*-value was calculated using the generalized estimating equation method

^b^The Student t test was used to analyze the differences between groups at indicated time points

### Hemodynamic changes during analgesia

During anesthesia, the patients’ heart rate (Fig. 1C) and SpO_2_ (Fig. 1D) did not change drastically, and there was no significant difference between groups at each assessment time point (GEE, *P* > 0.05). None of the patients in both groups experienced episode of hypoxemia, respiration depression and bradycardia. The systolic blood pressure during analgesia did not change drastically (Fig. 1E), and there was no significant difference between groups in the systolic blood pressure (Table 3, GEE, *P* > 0.05). No patients experienced arterial hypotension. Although GEE analysis found that nalbuphine had a slight decreased trend after infusion (GEE, *P* = 0.010), the diastolic blood pressure during the period was still within the normal range (Fig. 1F). In summary, the use of sufentanil and nalbuphine did not cause the respiration depression and drastic hemodynamic changes during analgesia in ICU patients.

### Analgesic and sedative effects of sufentanil and nalbuphine on different age groups

**Fig. 2:**
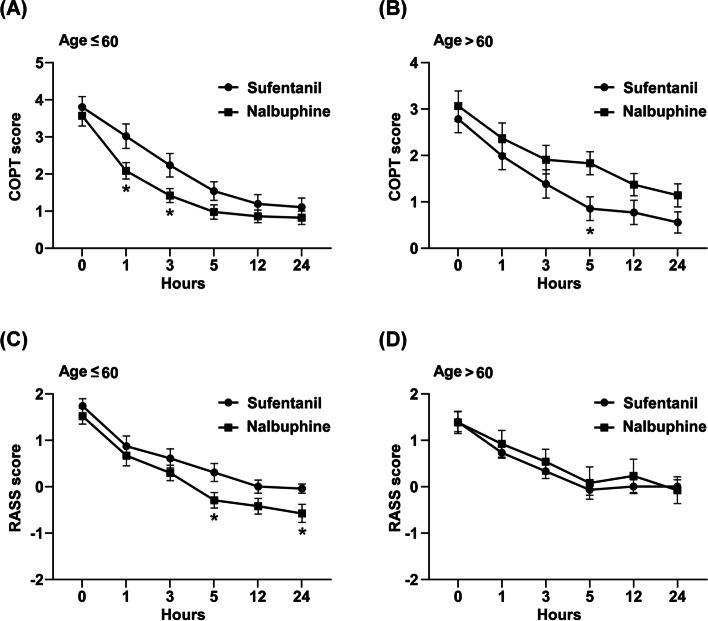
Age-stratified analyses of the analgesic and sedative effects of sufentanil and nalbuphine on ICU patients. Pain intensity in ICU patients who received nalbuphine or sufentanil at different time points under 60 (**A**) and over 60 years of age (**B**). Sedation/restlessness intensity in ICU patients who received nalbuphine or sufentanil at different time points under 60 (**C**) and over 60 years of age (**D**). Data were expressed as mean ± SD. The *P*-value was calculated using the generalized estimating equation method. The asterisk indicates that there is a significant difference between the groups at the specified time point

As shown in Fig. 2A, nalbuphine showed better analgesic effect for ICU patients under 60 years of age than sufentanil (GEE, *P* = 0.004). In contrast, sufentanil showed a better analgesic effect than nalbuphine for ICU patients over 60 years of age (Fig. 2B, GEE, *P* = 0.005). The CPOT score of the sufentanil group was significantly lower than that of the nalbuphine group at 5 h after infusion (0.87 ± 0.26 vs. 1.85 ± 0.25, *P* = 0.011). Similarly, nalbuphine has better sedative effect than sufentanil for ICU patients under 60 years of age (Fig. 2C, GEE, *P* = 0.003). The RASS score of nalbuphine was significantly lower than that of sufentanil at 5 h (-0.30 ± 0.17 vs. 0.3 ± 0.19, *P* = 0.022) and 24 h (-0.58 ± 0.19 vs. -0.04 ± 0.10, *P* = 0.022) after infusion. For ICU patients over 60 years old, there was no significant difference in the sedative effects between nalbuphine and sufentanil (Fig. 2D, GEE, *P* > 0.05).

## Discussion

In this study, the analgesic and sedative effects of sufentanil and nalbuphine was investigated for the first time in ICU patients. Our findings showed that both nalbuphine and sufentanil provided adequate analgesia. No patients in the ICU experienced hypoxemia, respiratory depression, arterial hypotension and bradycardia during analgesia and sedation with nalbuphine and sufentanil. Stratified analysis further showed that nalbuphine presented a better analgesic and sedative effects than sufentanil in ICU patients under 60 years of age. Moreover, sufentanil had a better analgesic effect than nalbuphine in ICU patients over 60 years of age. Thus, the results of this study suggest that nalbuphine can be regarded as a reasonable alternative for sufentanil to provide analgesia and sedation for ICU patients, especially for patients under 60 years of age.

Up to 70% of patients in the ICU suffer from moderate to severe pain intensity, and these experiences may leave long-term imprints, such as chronic pain and post-traumatic stress disorder [26, 27]. In a study of 599 survivals 6 months after discharge, 17% patients remember severe pain in the ICU, and 18% patients developed post-traumatic stress disorder [28]. Many patients still believe that the pain they experienced during ICU is considered to be the cause of sleep disturbance after discharge from the hospital [29]. In addition, inappropriate management in ICU may be resulted in hypoxemia, thromboembolic and pulmonary complications, increased ICU stay, pain-associated immunosuppression, and readmission [1, 30]. However, many patients in the ICU, especially those with aphasia, dementia, delirium or intubated and mechanically ventilated patients, cannot self-report their pain verbally. This is why we use CPOT instead of self-report to objectively measure the pain scores and the agitation or sedation levels in ICU patients.

Respiration depression has been the main factor restricting the use of opioids. Therefore, the clinical guidelines recommend the use of non-opioid analgesics to reduce or replace the use of opioids [8]. Previous studies demonstrated that the dose–effect curve of nalbuphine in respiratory depression is flatter than that of morphine, and nalbuphine dose greater than 0.15 mg/kg causes respiratory depression, with a ceiling effect dose of 30 mg/70 kg [21, 31]. In this study, the average infusion dose of nalbuphine was 0.165 ± 0.057 mg/kg, which was just the marginal dose that cause respiratory depression (Table 4). In addition, the infusion dose of nalbuphine at different time points remained stable (GEE, *P* > 0.05). Therefore, for the ICU patients in this study, the results that nalbuphine did not cause respiratory depression, hypoxemia and bradycardia may be attributed to the use of low-dose nalbuphine. In addition, supplemental oxygen in the ICU ward may also improve oxygenation in patients with reduced SpO_2_. These results also strengthen the safety of nalbuphine for analgesia in ICU patients.

**Table 4:** Infusion doses of sufentanil and nalbuphine in ICU patients at different time points

Infusion	Mean dose	**Infusion time**					
		1 h	3 h	5 h	12 h	24 h	*P*-value
Sufentanil (vg/kg)	0.280 ± 0.044	0.371 ± 0.144	0.262 ± 0.092	0.260 ± 0.084	0.267 ± 0.086	0.237 ± 0.074	0.951
Nalbuphine (mg/kg)	0.165 ± 0.057	0.170 ± 0.124	0.163 ± 0.127	0.163 ± 0.127	0.165 ± 0.131	0.164 ± 0.131	1.000

The *P-*value was calculated using the generalized estimating equation method

It is known that aging is related to the gradual decrease of the functional reserve of all organ systems, including the nervous system. With age, the concentration of neurotransmitters, norepinephrine and dopamine receptors, and nervous tissue mass and density gradually decreased, which ultimately affects the elderly’s pain perception and response to anesthetics [32, 33]. Therefore, advanced age is generally considered to be an independent factor affecting anesthesia/analgesia/sedation [32, 34]. The age stratified analysis in this study showed that nalbuphine has better analgesic and sedative effects than sufentanil in ICU patients under 60 years of age (Fig. 2). This result may be due to the different pharmacology of sufentanil and nalbuphine. Sufentanil is a high affinity μ-opioid receptor agonist and a selective κ-opioid receptor agonist [35]. In contrast, nalbuphine mixed agonist–antagonist properties, which mainly acts on κ-opioid receptors (analgesic), and processes opioid antagonist effect (morphine-reversal) at the μ-opioid receptor [36]. Although the receptor levels and the efficiency of signal transduction after receptor binding in the elderly are controversial, studies have shown that the number and binding levels of κ-opioid and μ-opioid receptors in elderly rats are greatly reduced [37, 38]. On the contrary, for patients over 60 years old, sufentanil has better analgesic effects than nalbuphine (Fig. 2). In this study, the difference between nalbuphine and sufentanil did not reach statistical significance at certain time points, which may be due to the small number of patients in the study. Therefore, future studies should have prospective designs and should recruit more ICU patients to explore the role of age confounder in the analgesic effect of nalbuphine.

## Limitation

This study has several limitations. Since this study focused on patients admitted to ICU, the small population size and the relative complexity of hospitalized diseases are limitations. Thus, the current results can only conclude that nalbuphine has a sustained and stable analgesic and sedative effect on ICU patients, but the results can not reflect other groups of patients who need analgesia. In addition, due to the limitations of retrospective study, this study lacks follow-up data. Therefore, we cannot assess patients’ satisfaction with nalbuphine and sufentanil in pain management while in the ICU, and understand the patients’ pressure disorders after discharge from the hospital. Future prospective studies with a larger sample size are required to reduce the limitations associated with the study.

## Conclusion

In comparison with sufentanil, nalbuphine showed a sustained and stable analgesic and sedative effect on ICU patients with mild to moderate analgesia needs. During analgesia, nalbuphine did not cause respiratory depression and drastic hemodynamic changes. For ICU patients under 60 years old, nalbuphine has better analgesic and sedative effects than sufentanil. Therefore, we suggested that nalbuphine can be a useful alternative to sufentanil for patients who are admitted to ICU and need analgesia, especially these under 60 years of age. Future studies should have prospective design and should focus on well-defined ICU patients to further confirm age effect between nalbuphine and sufentanil.

## Data Availability

The data used and analyzed in this study are available from the corresponding author upon reasonable request.

## Abbreviations

SpO_2_: Blood oxygen saturation
ICU: Intensive care unit
APACHE: Acute Physiology and Chronic Health Evaluation
CPOT: Critical Care Pain Observation Tool
RASS: Richmond Agitation-Sedation Scale
SD: Standard deviation
IQR: Inter-quartile range
